# Design of a novel instrument for active neutron interrogation of artillery shells

**DOI:** 10.1371/journal.pone.0188959

**Published:** 2017-12-06

**Authors:** Camille Bélanger-Champagne, Hannes Vainionpää, Pauli Peura, Harri Toivonen, Paula Eerola, Peter Dendooven

**Affiliations:** 1 Helsinki Institute of Physics, Helsinki, Finland; 2 JHV Physics, Riihimäki, Finland; 3 HT Nuclear, Hyvinkää, Finland; University of Liverpool, UNITED KINGDOM

## Abstract

The most common explosives can be uniquely identified by measuring the elemental H/N ratio with a precision better than 10%. Monte Carlo simulations were used to design two variants of a new prompt gamma neutron activation instrument that can achieve this precision. The instrument features an intense pulsed neutron generator with precise timing. Measuring the hydrogen peak from the target explosive is especially challenging because the instrument itself contains hydrogen, which is needed for neutron moderation and shielding. By iterative design optimization, the fraction of the hydrogen peak counts coming from the explosive under interrogation increased from $53_{-7}^{+7}$% to $74_{-10}^{+8}$% (statistical only) for the benchmark design. In the optimized design variants, the hydrogen signal from a high-explosive shell can be measured to a statistics-only precision better than 1% in less than 30 minutes for an average neutron production yield of 10^9^ n/s.

## 1 Introduction

Society faces many threats through the malicious use of CBRNE (Chemical, Biological, Radiological, Nuclear and/or Explosive) materials. The detection of illicit trafficking or other criminal acts, as well as many security and safety applications, call for novel material analysis techniques and instruments. These detection systems should be non-destructive but still be able to detect and identify the threat objects, even from inside a shielding or masking enclosure. Active interrogation methods that use penetrative particle beams can reveal the presence of CBRNE materials.

In prompt gamma neutron activation analysis (PGNAA), an unknown object is exposed to a high neutron flux and the outgoing prompt gamma radiation is measured with a high energy resolution gamma spectrometer [1]. The emitted gamma rays are isotope-specific, so PGNAA can be used to detect the presence of nearly all elements. The relative intensity of the gamma ray peaks in the energy spectrum can be used to measure the relative fractions of elements inside the unknown target. The prompt gamma-ray emissions can occur after neutron capture on the atomic nucleus or via inelastic scattering. Thermal neutrons are most likely to interact with the target via neutron capture. For an inelastic scattering event to result in gamma-ray emission, the interacting neutron must be a fast neutron with a kinetic energy larger than the energy of the gamma emission of the target isotope.

Applications of the PGNAA technique exist in many contexts, and specialized systems are designed based on the materials, elements and isotopes that must be identified in each application. Some luggage handling and landmine detection systems rely specifically on the use of thermal neutrons [2] while some cargo handling systems focus on the use of fast neutrons [3]. Neutron-based systems can also be used to detect special nuclear material [4].

The use of PGNAA for CBRNE safety applications is well established [5–7]. In the subset of applications that focus on military ordnance, explosives and chemical weapons are the principal identification targets inside unknown objects. The ratio of the main gamma-ray peaks of hydrogen and nitrogen can be used to identify the presence of high explosives. The gamma-ray signal from As, F, P, S and Cl is needed to identify chemical weapons [5], while the additional detection of bromine and iodine is desirable, according to the Organisation for the Prohibition of Chemical Weapons (OPCW) [8]. A few fully integrated commercial systems are available, such as Ortec PINS3-CF [9] and PINS3-CW [10] and EADS SODERN NIPPS [11]. These systems use different types of neutron sources: the PINS3-CF system uses a Cf-252 spontaneous fission source while the PINS3-CW and NIPPS systems use deuterium-deuterium fusion neutron generators. The algorithm used by the Ortec systems to identify explosives and chemical warfare agents (CWA) is described in [7]. These different source types produce neutrons with very different energy spectra. The impact of the source neutron spectrum has been studied for both safety applications with explosives and CWA [12] and for bulk sample characterisation instruments [13].

Isotope-specific prompt gamma radiation can arise from fast or thermal neutron interactions, depending on the material of interest. Therefore, time separation of the fast neutron-induced prompt gamma signal and the thermal neutron-induced prompt gamma signal reduces the background in both measurements. The existing instruments that use continuous sources cannot separate the two prompt gamma signals. Of the existing instruments, according to their technical specifications, only the EADS SODERN NIPPS uses signal type separation, but between prompt and delayed gamma emissions. The neutron generator is operated for a preset number of pulses and then data acquisition continues during a longer period after the last pulse [11]. Two spectra are collected, a prompt gamma spectrum for the duration of the preset pulses, and a delayed gamma spectrum during the long pause. The two prompt gamma-ray contributions, from fast neutron- and thermal neutron-induced reactions, are collected together in the prompt spectrum. The delayed gamma spectrum arises from radioactive decays after neutron activation of isotopes in the material, independently of the process that activated the material.

This article presents results of Monte Carlo simulations that were used to design a new PGNAA instrument. The design studies focus on the detection of explosives inside an artillery shell. While CWA and smoke agents have specific elemental markers like Cl, P, F, etc., high explosives are all largely made up of a combination of H, C, N and O [6], which are also present in everyday materials. High explosive identification relies on the measurements of elemental ratios of these elements, where separation of the signal from background sources is challenging. In the proposed instrument, the neutrons are generated using the deuterium-deuterium fusion (D-D) reaction. The most relevant prompt gamma-ray emissions for the elements of interest are listed in Table 1, along with signature elements of CWAs. The inelastic scattering emissions from carbon and oxygen are not accessible using the 2.5 MeV neutrons from the D-D reaction. The hydrogen and nitrogen emission from thermal neutron capture are accessible. The main focus is thus to measure the H/N ratio with high accuracy and precision, which can provide discrimination as to the type of explosive present. If the H/N ratio can be determined with a precision better than 10%, the most common high explosives in Table 2 can be uniquely identified. In turn, this requires accurate and precise measurement of the H and N components of the prompt emission gamma-ray spectrum from the explosive material. A precisely pulsed neutron generator is used. The timing profile and data collection in different time windows relative to the beginning of each pulse are chosen to maximise isotope identification efficiency by separating the fast- and thermal-neutron induced signals during each pulse cycle [14, 15]. The neutron emission rate can be very large (>>10^8^ n/s for typical generators) when compared with portable spontaneous fission neutron sources or sources containing an alpha emitter inside a low-Z element matrix (such as americium-beryllium). Furthermore, they do not contain fissile material and they can be switched completely off when not in operation.

**Table 1:** Selected gamma-ray emission lines for the detection and identification of high explosives and CWA [5, 16, 17].

*High Explosives*		
**Isotope**	**Gamma ray energy [MeV]**	**Process**
^1^H	2.22	thermal neutron capture
^12^C	4.43	neutron inelastic scattering
^14^N	10.8	thermal neutron capture
^16^O	6.13	neutron inelastic scattering
*Chemical Warfare Agents*		
^19^F	1.24, 1.35, 1.36	neutron inelastic scattering
^31^P	0.63, 1.07	thermal neutron capture
^31^P	1.27, 2.23	neutron inelastic scattering
^32^S	0.84, 3.2	thermal neutron capture
^35^Cl	0.52, 0.79, 1.16, 1.95	thermal neutron capture
^75^As	6.29, 6.81, 7.02	thermal neutron capture
^75^As	0.57	neutron inelastic scattering

**Table 2:** Elemental ratio H/N for some common high explosives [18].

Explosive	Elemental ratio
TNT	1.67
RDX	1.0
Comp. B	1.25
ANFO	2.2

## 2 Materials and methods

**Fig 1:**
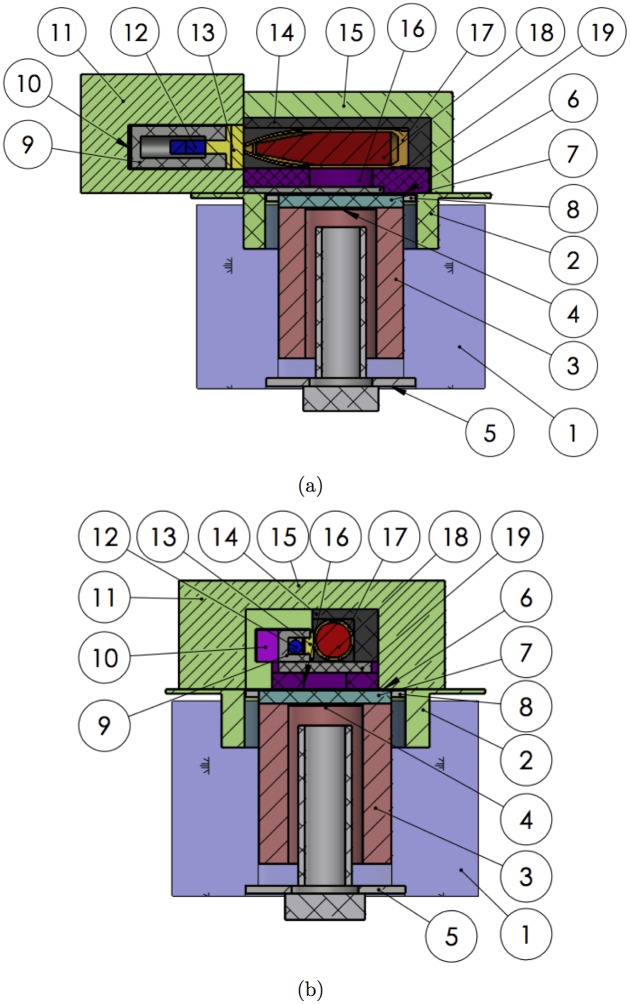
Instrument geometries. Cross-section drawings of the optimized geometries of the proposed instruments for a case with (a) neutron-sensitive detectors (“shell-point” design) and (b) detectors that are not affected by the presence of a large neutron flux (“shell-side design”). See Table 3 for the list of numbered parts and Table 4 for their dimensions.

The benchmark scenario for this study is the case where an old artillery shell is recovered but the content (chemical agent, conventional explosive, incendiary, smoke, practice round) is unknown. The object is interrogated via neutron activation analysis. There are two different design concepts for the apparatus in this study, depending on the characteristics of the detector it contains. The optimized instrument designs that were obtained in this study are shown in Fig 1.

The neutron source is a pulsed generator that uses the deuterium-deuterium fusion reaction to generate an intense pulsed flux of 2.5 MeV neutrons. A significant fraction of these initial neutrons is slowed down to the thermal energy range at the output of the generator via the use of a neutron moderator material.

The object under study is placed in an interrogation cavity surrounded by a neutron reflector for maximum interrogation efficiency. A high energy resolution gamma-ray detector is located next to the interrogation cavity, at a 90° angle to the accelerator line of the neutron generator. As an example, a cerium-doped lanthanum bromide detector in a commercially available size is used. Such detectors have shown potential to be used in similar CBNRE detection applications [19]. The detector is enclosed in both neutron and gamma-ray shielding, retaining only a window looking at the object under interrogation. The entire experimental setup is surrounded by shielding materials to ensure the safety of the operation personnel. In Fig 1(a), the gamma-ray detector is aligned with the point of the artillery shell under interrogation and it is located relatively far from the main neutron flux. This is the reference design for use with a neutron-sensitive detector, which will be referred to as the “shell-point” design in the rest of this paper. In Fig 1(b), the gamma-ray detector is pointed at the side wall at the centre of the artillery shell and is much closer to the main neutron flux and the unknown object. This is the reference design for an apparatus that uses a detector that is not unduly affected by the presence of neutrons and can thus be located closer to the gamma-ray emission volume within the unknown object, which we call the “shell-side” design. Thus, this configuration benefits from a significant increase in solid angle coverage for the gamma-ray detector.

### 2.1 GEANT4 configuration

All data samples used in this paper were simulated using Monte Carlo methods in the GEANT4 [20] software package, version 4.10.01p01. The data samples where simulated using the QGSP_BERT_HP built-in physics list for GEANT4 in order to provide accurate results for both electromagnetic and hadronic processes, and in particular for low-energy neutron physics. Neutron interaction data was taken from G4NDL4.5 [17], except in the case of ^113^Cd. We observed that, by default, models and data included in GEANT4 resulted in a poor match with the tabulated data for gamma emissions from thermal neutron capture on ^113^Cd [21]. So, for this isotope, we wrote custom gamma-emission data files based on [21] for use with GEANT4.

The instrument’s geometry is imported into GEANT4 from a CAD drawing using CADMesh [22]. The neutron generator’s ion source and accelerating cavity are omitted from the simulation. Each volume of the drawing is assigned a constituent material through which particle transport and interactions are calculated by GEANT4. The volumes and materials for the designs of Fig 1 are given in Table 3. The table lists the materials used at the start of the study as well as those selected after the optimization of the instrument design. Part #19 was not in the starting design and the volume it occupies in Fig 1 was included in part #16. The final-design dimensions of the components of the shell-point instrument are given in Table 4.

**Table 3:** List of volumes represented in GEANT4 for the instruments under study, along with their material composition at the start of the optimization studies and after completion of the study.

#	Name	Starting Material	Final design material
*Neutron generator and shield*			
1	Water tank	Water	Borated water (5% mass)
2	Water tank structural panel	Borated polyethylene (5% mass)	Borated polyethylene (5% mass)
3	Oil tank	C_5_H_12_O_4_	Borated C_5_H_12_O_4_ (5% mass)
4	Neutron generator target	Copper	Copper
5	Ion source structural ring	Stainless steel	Stainless steel
6	Neutron generator window	Aluminium	Aluminium
7	Main neutron moderator	Polyethylene	Polyethylene
8	Neutron generator casing	Stainless steel	Stainless steel
*Gamma detector and shield*			
9	Detector bunker	Lead	Lead
10	Detector neutron shield	Cadmium	Cadmium
11	Detector exterior shield	Borax decahydrate	Borax decahydrate
12	Gamma detector	LaBr_3_:5%Ce	LaBr_3_:5%Ce
13	Detector bunker window	Li_2_CO_3_	Li_2_CO_3_
*Unknown object and cavity*			
14	Object cavity neutron reflector	Graphite	Graphite
15	Object cavity exterior shield	Borax decahydrate	Borax decahydrate
16	Object cavity floor	Polyethylene	Graphite
17	Artillery shell casing	Iron	Iron
18	Artillery shell contents	TNT	TNT
19	Shielding floor plate	—	Lead

Parts numbers in the first column correspond to those of Fig 1.

**Table 4:** List of volumes represented in GEANT4 for the instruments under study, along with their final dimension at the end of the study, for the “shell-point” design.

#	Name	Shape	Dimensions [mm] (height×width×length or height×diameter)
*Neutron generator and shield*			
1	Water tank	rectangular prism	730×970×1150
2	Water tank structural panel	rectangular prism†	220×1000×1200
3	Oil tank	hollow cylinder	600×500(outer)×280(inner)
4	Neutron generator target	cylinder	10×280
5	Ion source structural ring	ring	30×600(outer)×250(inner)
6	Neutron generator window	cylinder	6×600
7	Main neutron moderator	cylinder	50×500
8	Neutron generator casing	ring	25×600(outer)×500(inner)
*Gamma detector and shield*			
9	Detector bunker	cylinder†	400×208
10	Detector neutron shield	cylinder†	406×212
11	Detector exterior shield	rectangular prism†	486.5×650×615
12	Gamma detector	cylinder	140×50.8
13	Detector bunker window	cylinder†	121×215
*Unknown object and cavity*			
14	Object cavity neutron reflector	rectangular prism†	200×749×300
15	Object cavity exterior shield	rectangular prism†	405×838×580
16	Object cavity floor	rectangular prism with circular hole†	100×745×300 250 (hole diameter)
17	Artillery shell casing	cylinder†	655×154.4
18	Artillery shell contents	cylinder†	540×132
19	Shielding floor plate	rectangular prism	20×590×300

Parts numbers in the first column correspond to those of Fig 1. For simplicity, the shape and dimensions of an enclosing shape are listed for parts marked with †.

All the simulated data samples used in these studies are built from single neutron events. The neutrons are generated isotropically and uniformly over a disk of 5 cm in diameter at the centre of the beam-facing side of the copper target disk of the instrument’s neutron generator. All neutrons are generated with an initial energy of 2.5, as would result from the deuterium-deuterium fusion reaction in the neutron generator [23]. All neutrons are created in the simulation at time *t* = 0 within their event. The simulated neutrons behave like a generator pulse with a time spread of zero. The path of each initial neutron, and its interactions with the material of the experimental apparatus, are tracked by GEANT4 until the neutron is captured or escapes outside of the world volume of the simulation (a 2x2x2 m^3^ air-filled cube with the simulated instrument at the centre). All secondary particles created by interactions in the world volume are also tracked. In the neutron flux optimisation studies described in Section 3.1, the sample size is 25 million initial neutrons. For gamma-ray flux optimisation studies described in Section 3.2, the sample size is 300 million initial neutrons.

## 3 Results

### 3.1 Optimization of neutron flux

**Fig 2:**
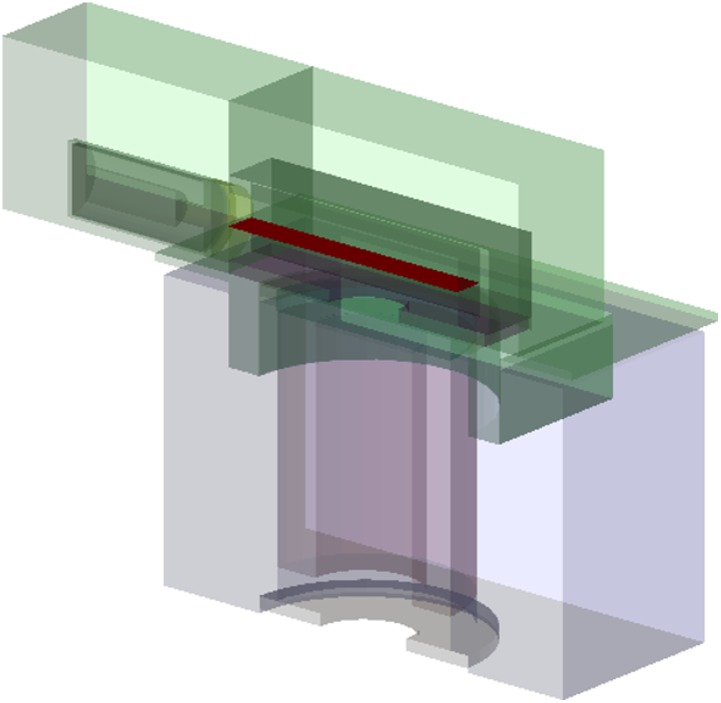
GEANT4 model of the instrument. GEANT4 cutaway image of the initial geometry used to study neutron flux characteristics in the unknown-object cavity. The shell-point design is used, with the detector and artillery shell omitted. The neutron scoring plane is shown in solid red.

The characteristics of the neutron flux in the unknown-object cavity depend mainly on the geometry and material characteristics of the main neutron moderator, the cavity floor and the neutron reflectors. They are however not strongly affected by the details of the detector assembly and shielding. So, to study and optimise the neutron flux, we generated data samples based on our initial design geometry of the shell-point instrument design. The representation of this starting geometry within the GEANT4 simulation is shown in Fig 2. We did not include the ammunition shell object in the cavity. To record the neutron flux characteristics in the cavity where the object would be located, we defined an infinitesimally thin scoring plane at the centre of the unknown-object cavity (shown in red in Fig 2) and recorded the energy and time distributions of the neutrons crossing this plane. This gives us a very good estimate of the neutron flux onto the unknown object that is independent of the geometry and material composition of the unknown object.

The design item to be optimised is the size and thickness of the neutron moderator material, which is shown as part #7 in Fig 1. In the instrument, this piece plays a double role as the main neutron moderator and as electrical insulation at the end of the neutron generator. This double role puts some constraints on the design choices: the material chosen must be an electrical insulator, and the shape is constrained to be a disk of diameter matching that of the neutron generator. Polyethylene was chosen as the moderating material, for its low cost, high moderating power, ease of machining and electrical properties. The main geometrical parameter to optimise is the thickness of the moderator disk. A thicker moderator is expected to thermalize a larger fraction of the fast neutron flux. However, it will also attenuate the overall neutron flux through the unknown object via scattering and absorption. As the moderator is structural in the instrument, its thickness also affects the distance between the neutron generation disk and the scoring plane and introduces a geometric distance effect into the observed neutron flux. We simulated data samples in GEANT4 varying the thickness of the main moderator material, using thicknesses of 50, 70, 90 and 120 mm. A minimum thickness of 50 mm is needed for adequate electrical insulation.

**Fig 3:**
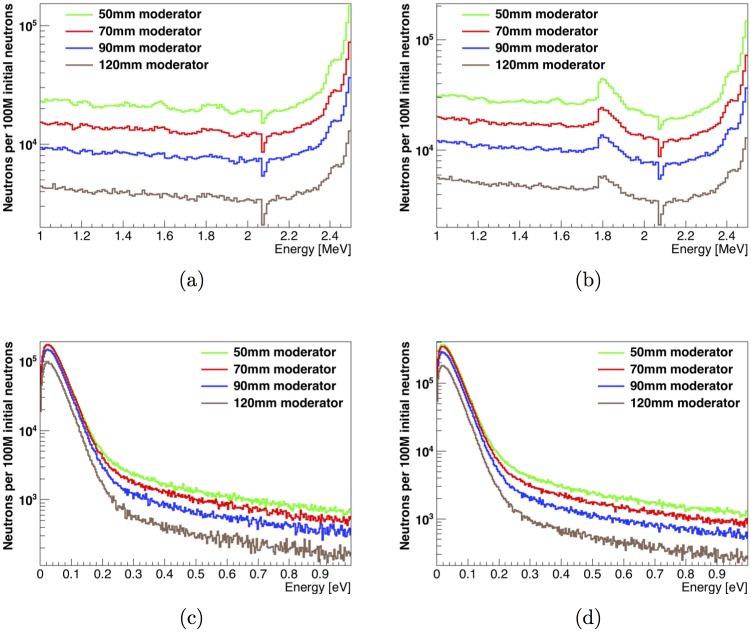
Neutron energy distributions in the unknown-object cavity with varying moderator thicknesses. Effect of the thickness of the polyethylene moderator on the energy distribution of (a) direct fast neutrons, (b) integrated fast neutrons, (c) direct thermal neutrons and (d) integrated thermal neutrons in the unknown-object cavity. A carbon elastic scattering resonance can be observed just below 2.1 MeV. In (b), the broad, asymmetrical peak between 1.8 and 2 MeV is due to neutrons reflected by the graphite shield located around the unknown-object cavity, shown as part #14 in Fig 1.

The energy distributions of fast (defined here to be within the energy window between 1 and 2.5 MeV) and thermal (defined here to have an energy less than 1 eV) neutrons in the unknown-object cavity for the tested moderator configurations are shown in Fig 3. For each neutron energy range, we define 2 types of distributions. “Direct” neutron distributions record the energy of each neutron the first time it crosses the neutron scoring plane at the centre of the unknown-object cavity. “Integrated” neutron distributions record the energy of all instances of a neutron crossing the scoring plane, including multiple entries per neutron if the neutron is backscattered toward the scoring plane one or more times. The deficit of neutrons below 2.1 MeV in the fast neutron spectra is due to a carbon elastic scattering resonance at 2.075 MeV [17]. The broad, asymmetrical peak between 1.8 and 2 MeV in the integrated fast neutron distribution is due to the presence of neutrons reflected by the graphite shield located around the unknown-object cavity. The main effect of the change of moderator thickness is on the number of fast neutrons, which decreases steadily with increased moderator thickness. The number of thermal neutrons present in the unknown-object cavity also decreases with increased moderator thickness, but not as strongly.

**Fig 4:**
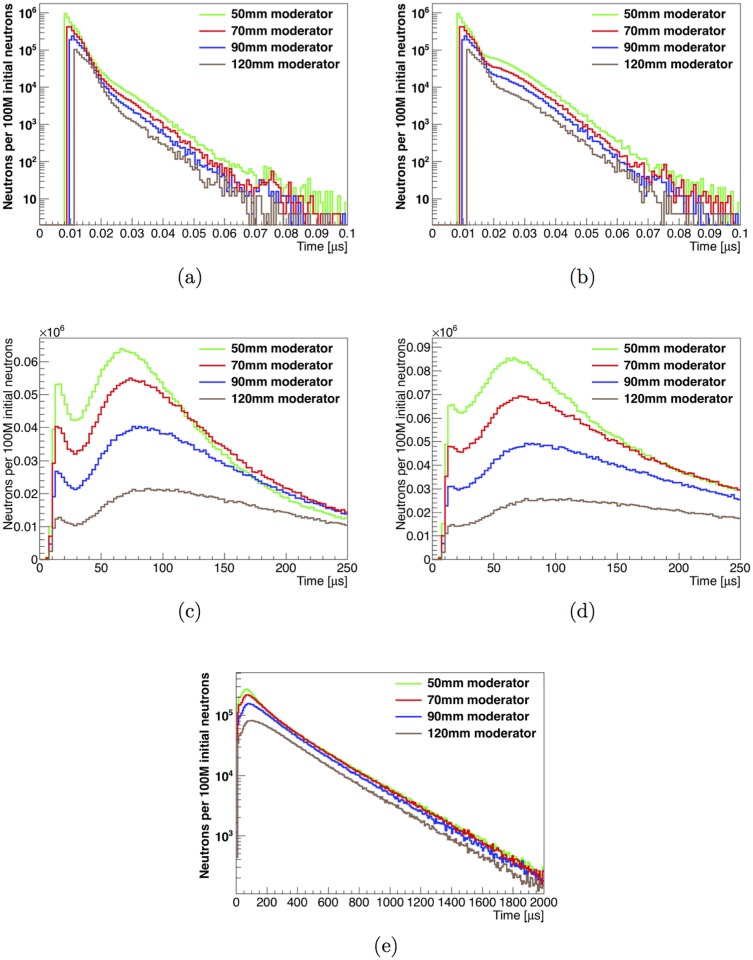
Neutron time distributions in the unknown-object cavity with varying moderator thicknesses. Effect of the thickness of the polyethylene moderator on the time distribution of (a) direct fast neutrons, (b) integrated fast neutrons, (c) direct thermal neutrons and (d-e) integrated thermal neutrons in the unknown-object cavity, shown for two different time ranges.

The time distributions of fast (1-2.5 MeV) and thermal (<1 eV) neutrons in the unknown-object cavity for the tested moderator configurations are shown in Fig 4. The start of the time distribution of fast neutrons changes with moderator thickness, and is consistent with the time-of-flight of 2.5 MeV neutrons between the generation plane and scoring plane as the distance increases between the neutron generation target and the unknown-object cavity. The integrated fast-neutron distribution has a larger contribution at longer times relative to the direct-neutron time distribution, but in both cases >99.9% of the fast neutrons travel through the cavity within the first 0.1 μs of the neutron’s lifetime. The time distribution of thermal neutrons rises fast starting around 10 μs and extends up to a few milliseconds, as shown in Fig 4(e). It is important to note that this means the thermal neutron survival time is longer than the neutron generator’s period when operating at the design frequency of at least 1 kHz. The direct thermal neutron distribution features a minimum around 30 μs and a broad peak at 70–90 μs, depending on moderator thickness. The integrated distribution is somewhat broader, with a high multiplicity of recorded neutron crossings until around 100 μs.

We selected 50 mm of polyethylene as the design variant that provided the highest overall neutron flux, while generating a sufficient amount of thermal neutrons. As the moderator layer also acts as an electrical insulator, the thickness cannot be decreased further.

#### 3.1.1 Effect of the moderator volume on the thermal neutron flux

**Fig 5:**
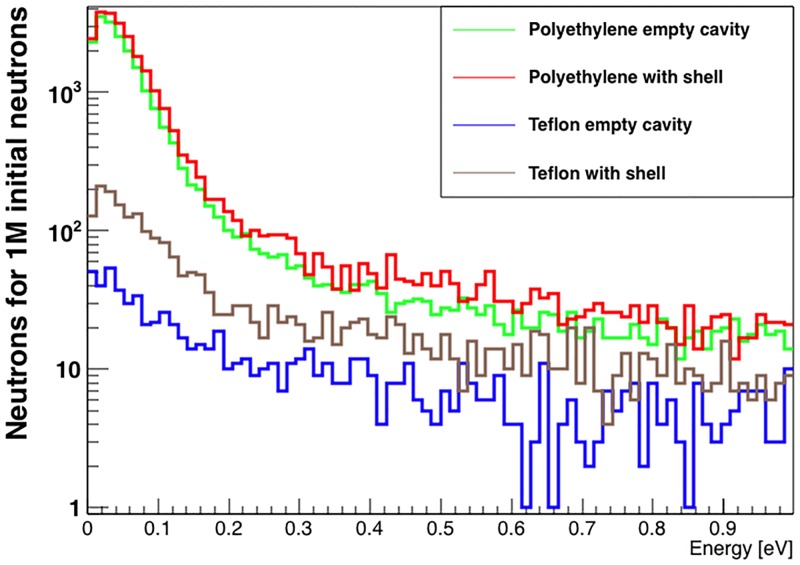
Energy distribution of the integrated thermal neutrons at the centre of the unknown-object cavity for different moderation scenarios. Two distributions are the result of simulations where the moderator volume is made up of polyethylene, with (red) and without (green) an artillery shell in the cavity. The other two distributions use Teflon in the moderator volume, with (brown) and without (blue) an artillery shell in the cavity.

To emphasize the importance of the neutron moderator in providing a significant thermal neutron flux in the instrument’s cavity, we simulated 4 samples of 1 million initial neutrons in the final instrument design. In the 4 samples, the cavity is either empty or filled with a TNT ammunition shell, and the moderator volume is either polyethylene (an efficient moderator and our material of choice) or Teflon, a poor neutron moderator. The energy distribution of integrated thermal neutrons crossing at the centre of the cavity for the 4 scenarios is shown in Fig 5. The self-moderating power of the artillery shell is clearly visible when comparing pairs of distributions with the cavity empty and filled for the same material in the moderator volume. The presence of the polyethylene moderator volume results in 10.4 times higher thermal neutron flux at the centre of the shell-filled cavity, compared to Teflon.

### 3.2 Gamma-ray emission and detection

After optimization of the neutron flux to the unknown-object cavity, data samples of 300 million initial neutrons were generated to study the details of gamma-ray emission by the reference object (a TNT-filled iron artillery shell) and gamma-ray flux to the gamma detector. The uncertainty on all results shown is only the statistical uncertainty from the Monte Carlo statistics, unless stated otherwise.

**Fig 6:**
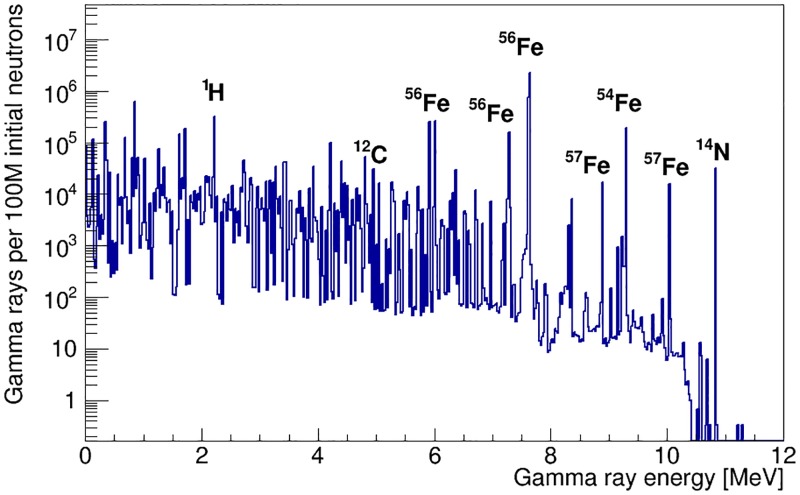
Neutron-activated gamma-ray emission spectrum of a TNT-filled iron artillery shell. The spectrum is obtained using the instrument configuration shown in Fig 1(a), after optimisation of the neutron flux. Some of the highest-intensity peaks are labelled with their isotopic source.

Fig 6 shows the simulated neutron-induced gamma-ray emission spectrum of an iron artillery shell filled with TNT. The shell-point instrument configuration, shown in Fig 1(a), was used, with a 50 mm thick neutron moderator. This configuration is used to illustrate all results in this section, but they are general results that also apply to the configuration shown in Fig 1(b).

**Fig 7:**
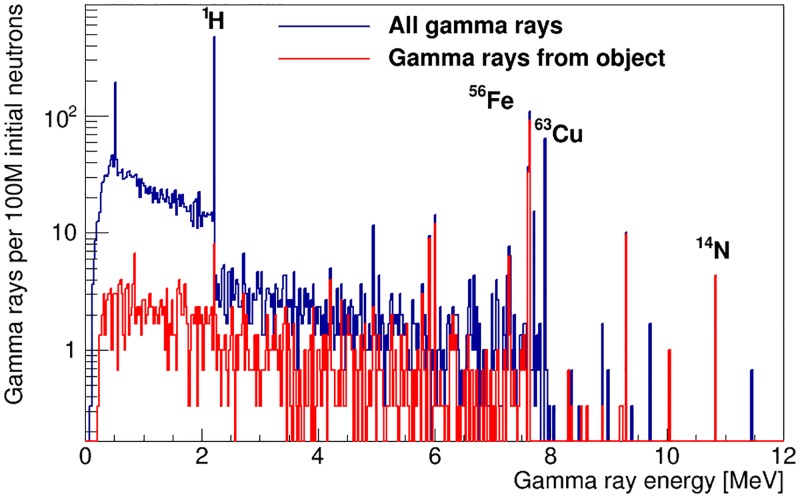
Energy spectrum of gamma rays incident on the active detector material in the initial design of the shell-point instrument configuration. The total incident spectrum is shown in blue, while gamma rays from the artillery shell and TNT payload are shown in red. The most important peaks for identification of explosives, H and N, are labelled, as well as other large peaks from iron in the shell and copper in the neutron generator target.

The emitted gamma rays are transported through the simulated instrument by GEANT4, along with all gamma-ray emissions occurring in other volumes of the shell-point experimental setup. Fig 7 shows the energy spectrum of the incident gamma rays, from all materials and all volumes in the experimental setup, on a cylindrical detector of 5 cm in diameter and 7.5 cm in length. It also shows the signal contribution from the simulated artillery shell. Detector efficiency and resolution effects are not folded into the spectra.

The 2.22 MeV gamma rays thermal-neutron capture on hydrogen originate predominantly from sources other than the artillery shell. We define the hydrogen signal fraction *SF_H_* of gamma rays incident on the detector as:

$$\begin{array}{c} SF_{H}=\frac{\text{Number of gamma rays from unknown object}}{\text{Total number of gamma rays}} \end{array}$$ (1)

in the gamma-ray energy range 2.16–2.28 MeV. The value of *SF_H_* is a measure of the explosive-identification capability of the instrument. The design of the instrument was modified in successive design steps to maximize the value of *SF_H_*. A larger value of *SF_H_* means that fewer total counts are necessary, in the 2.16–2.28 MeV energy bins of the gamma-ray spectrum, to ascertain the presence of hydrogen in the unknown object or precisely measure the number of counts from the unknown object.

**Fig 8:**
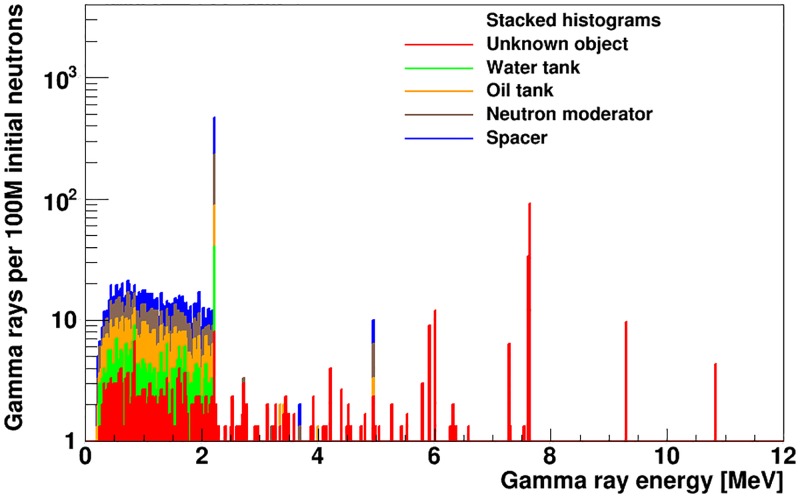
Number of gamma rays incident on the detector originating from each simulated volume that contributes significantly to the hydrogen gamma ray background, as a function of gamma ray energy. The histograms are drawn in a stack such that each histogram presents the contribution of a given volume on top of all volumes drawn below it.

In the gamma-ray energy spectra shown in Fig 7, $SF_{H}=2.8_{-0.4}^{+0.5}$%. For our simulated sample, this is comparable to the size of the Poisson uncertainty on the number of counts in the gamma-ray energy range of interest. Thus, the H/N ratio could not be used to identify explosive material for a real data sample of equivalent statistical power. Fig 8 shows the gamma-ray spectrum from the volumes that contribute significantly to the background hydrogen gamma rays in the shell-point configuration. The main background contributions are observed to arise from the shielding water tank, the polyethylene neutron moderator and the polyethylene floor of the cavity.

#### 3.2.1 Material composition

**Fig 9:**
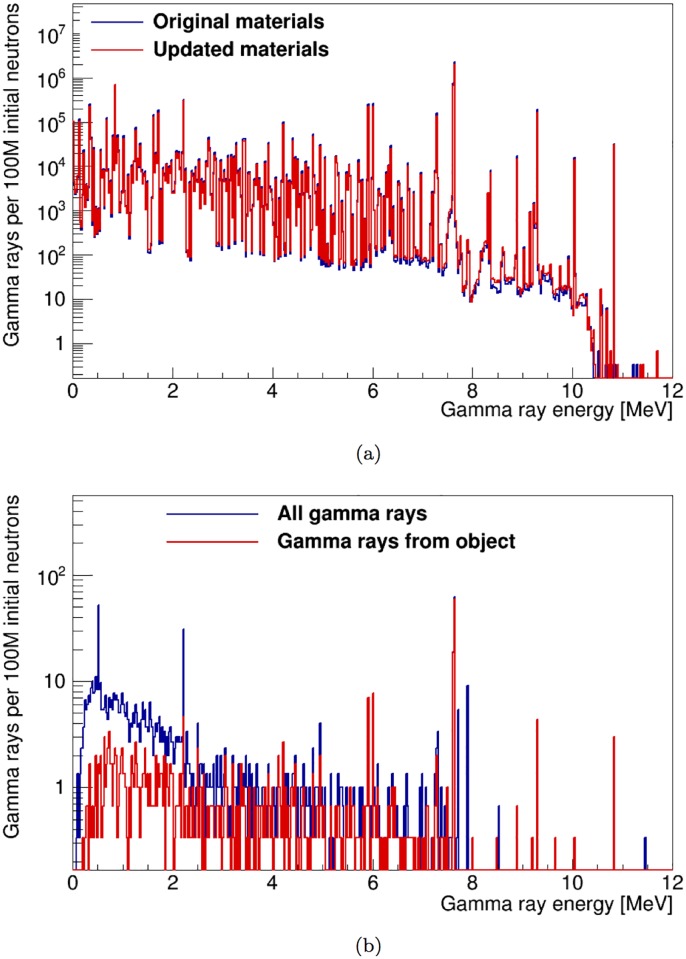
Number of gamma rays (a) emitted by the artillery shell and TNT payload and (b) incident on the detector after material substitutions in the instrument design.

To reduce the intensity of the 2.22 MeV hydrogen-capture gamma rays from sources other than the unknown object, material substitutions were made to the main contributing volumes, where they did not affect the basic functionality of the experimental setup. The material of the cavity floor was changed from polyethylene to graphite and we added 5% by mass boron, as a thermal neutron absorber, to the water and oil tanks. This also reduces the Compton background from hydrogen of other signal peaks located below 2.22 MeV. As is shown in Fig 9(a), the gamma-ray emission spectrum of the artillery shell is not affected by the material changes. Fig 9(b) shows the effect of the change on the energy spectrum of the gamma rays incident on the detector. The value of *SF_H_* obtained from the gamma-ray spectra of Fig 9(b) is $19_{-4}^{+4}$%, an improvement of close to an order of magnitude relative to Fig 7. The polyethylene neutron moderator is the only volume where the material can’t be changed without affecting the instrument’s performance.

#### 3.2.2 Lead shielding for gamma detector

**Fig 10:**
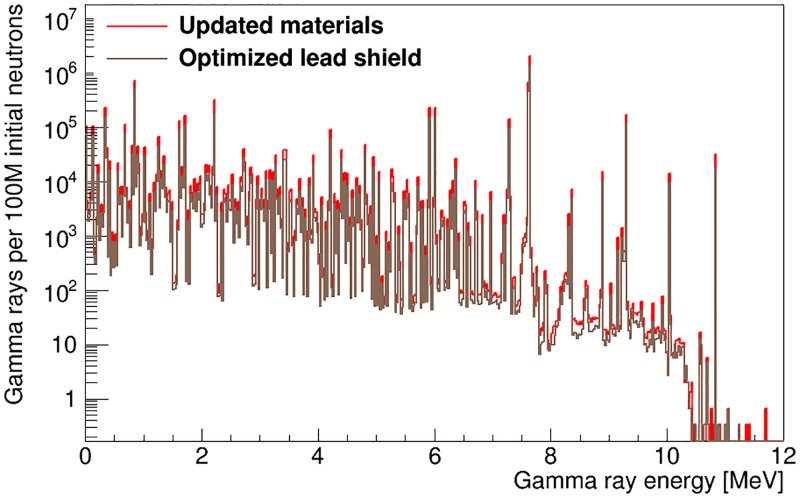
Number of gamma rays emitted by the artillery shell and TNT payload after updating material choices in the instrument design and after adding lead shielding.

**Fig 11:**
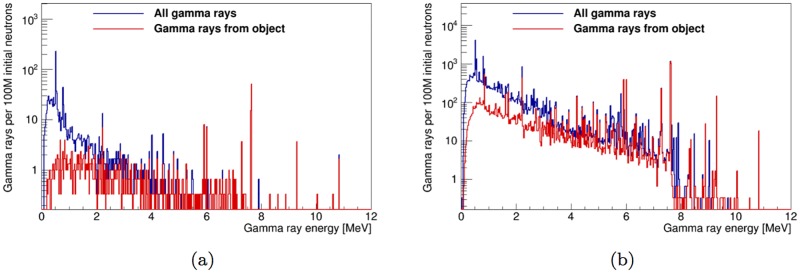
Energy spectrum of the gamma rays incident on the detector in the fully optimized instrument design. The total incident spectrum is shown in blue, while gamma rays from the artillery shell and TNT payload are shown in red. The spectra for the shell-point configuration of Fig 1(a) are shown in (a) while the spectra for the shell-side configuration of Fig 1(b) are shown in (b).

To suppress the background contribution from hydrogen in the polyethylene volume of the main neutron moderator, we introduced a new lead volume that completely shadows the gamma detector from straight-line emissions from the neutron moderator, shown as part #19 in Fig 1. We also increased the thickness of the lead bunker around the gamma detector (part #9 in Fig 1) on all sides except in the direction facing the unknown-object cavity. The effect of the inclusion of the modified lead shielding on the gamma-ray emissions of the artillery shell and the gamma-ray flux incident on the detector is shown in Figs 10 and 11(a). The presence of the lead plate results in a small attenuation of the neutron flux, which in turn reduces the number of gamma rays emitted by the artillery shell by 3%, uniformly over the whole gamma-ray energy range, see Fig 10. It also efficiently shields the gamma rays emitted inside the copper target of the neutron generator. The positioning of the new and modified lead components relative to hydrogen gamma-ray sources improves the hydrogen signal fraction to $SF_{H}=53_{-7}^{+7}$%.

### 3.3 Gamma-ray flux in optimized instrument geometry

After full design optimization of the instrumental apparatus, the gamma-ray flux onto the detector is shown in Fig 11 for both experimental configurations under study (see Fig 1). The dramatic difference in total gamma-ray flux is primarily due to the difference in the distance between the object under investigation and the gamma-ray detector between the two geometries. For the shell-point design, gamma rays account for 2.12±0.04% of the total particle flux on the detector volume, while in the shell-side configuration they account for 5.67±0.02%. Neutrons make up the rest of the flux, and the total flux on the detector in the shell-side configuration is 14 times larger than in the shell-point configuration. Thus, this configuration is only suitable if the gamma-ray detector used is unaffected by the presence of a large neutron flux and if the data acquisition chain can tolerate the high count rate. In the optimized shell-side configuration, $SF_{H}=45.9_{-0.8}^{+0.8}$%.

### 3.4 Effect of timing on observed gamma flux

**Fig 12:**
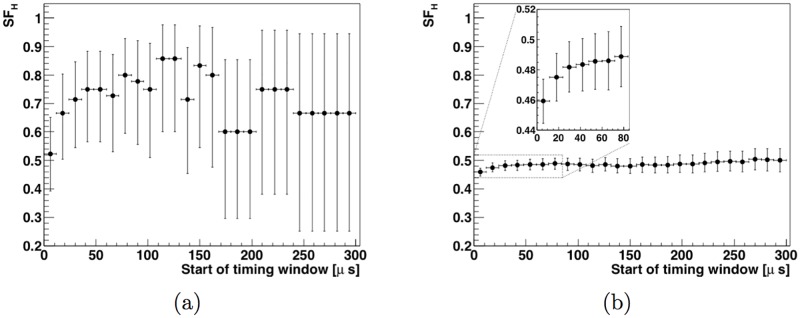
Hydrogen signal fraction *SF_H_* as a function of the start time of the thermal-neutron only timing window. The shell-point instrument design is shown in (a) and the shell-side instrument design is shown in (b). The error bars correspond to the 1*σ* statistical uncertainty resulting from the size of the Monte Carlo simulated data samples.

The gamma-ray energy spectrum can be split into a fast neutron-induced spectrum and a thermal neutron-induced spectrum to enhance the isotope identification power. As can be seen in Fig 4, the fast neutrons travel through the cavity within the first 0.1 μs after their creation, while the first thermal neutrons reach the cavity after around 10. A fast neutron-dominant window can be defined as *t*<1 μs from neutron creation, where only thermal neutrons from the previous pulses are present in the cavity. The neutron generator that will be used for the instrument has a pulse fall-off time in the range 10–30 μs [23]. Thus, in the simulated data, we can define a the time window *t*<30 μs from neutron creation, containing all the fast neutrons and a significant contribution of thermal neutrons, to provide an approximate representation of the neutron flux and gamma emissions in the instrument as the pulse switches off. A thermal-only time window can be defined with a starting time larger than 30 μs, and ending when the next pulse starts. Fig 12 shows the hydrogen signal fraction *SF_H_* as a function of the start time *t_s_* of a time integration window for both instrument configurations. The window goes from *t_s_* to 1 ms, corresponding to a neutron generator pulsing frequency of 1. This can be used to select the optimal boundary for the thermal-only time region. The value of *SF_H_* reaches a plateau at around *t_s_* = 50 μs in both configurations.

**Fig 13:**
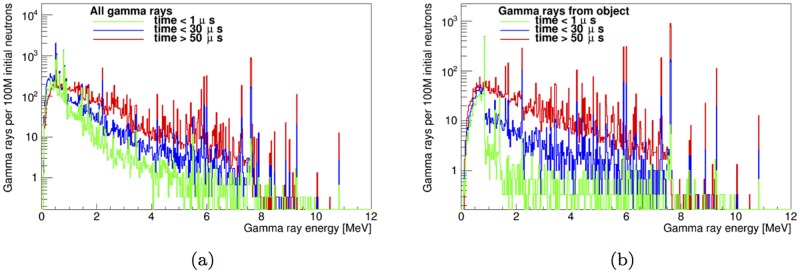
Energy spectrum of gamma rays incident on the detector for the time windows *t* <1 μs, *t* <30 μs and *t* >50 μs. The fully optimized instrument design in the shell-side configuration is used. The total gamma ray spectrum is shown in (a), while gamma rays from the artillery shell and TNT payload are shown in (b).

**Fig 14:**
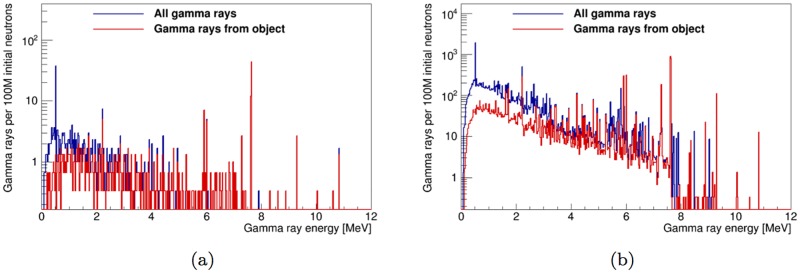
Energy spectrum of the gamma rays incident on the detector in the fully optimized instrument design in the thermal-only time window. The total incident spectrum is shown in blue, while gamma rays from the artillery shell and TNT payload are shown in red. Spectra for the shell-point configuration of Fig 1(a) are shown in (a) and spectra for the shell-side configuration of Fig 1(b) are shown in (b).

Thus, three time regions of interest are identified: *t*<1 μs, *t*<30 μs and *t*>50 μs. For the shell-side instrument configuration, the total gamma-ray spectrum on the gamma-ray detector and the spectrum from the artillery shell only are shown in Fig 13, for those three time windows. The energy spectra of gamma rays incident on the detector in the thermal neutron-only window for both design configurations is shown in Fig 14.

### 3.5 Effect of detector resolution

**Fig 15:**
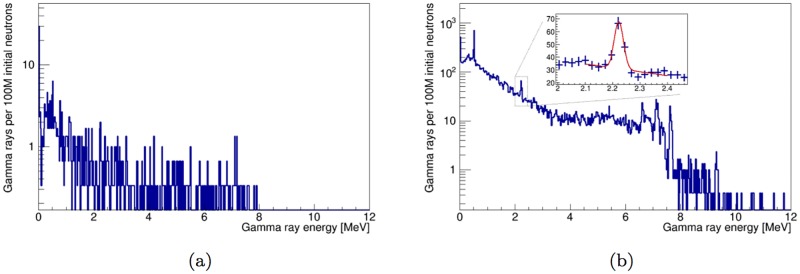
Spectrum of the energy deposited by all incident particles in a 2×3 inch LaBr_3_ detector in the fully optimized instrument design in the thermal-only time window. The simulated energy deposits are smeared using the energy resolution characteristics of [24]. Spectra for the shell-point configuration of Fig 1(a) are shown in (a) and spectra for the shell-side configuration of Fig 1(b) are shown in (b). The inset in (b) shows the peak from the hydrogen 2.22 thermal capture line. The result of a Gaussian peak fit over linear background is shown in red.

A realistic gamma-ray energy spectrum was obtained by a Gaussian smear of the spectrum of simulated detector hits in the thermal-only time window for a LaBr_3_:5%Ce detector. We used the manufacturer-specified energy resolution of a BriLanCe 380 detector from Saint-Gobain Crystals [24]. Hits from neutrons, gamma rays and secondary particles are included in these energy spectra, which are shown in Fig 15.

In the shell-side configuration, the peak area for the 2.22 hydrogen peak is obtained from the fit of a Gaussian peak over a linear background. The fit result is shown in the inset of Fig 15(b). The obtained peak area is 64±11 counts per 10^8^ initial neutrons. The limited statistics from the simulation of the shell-side configuration are not sufficient to perform a similar fit on the spectrum of Fig 15(a). By combining the result of the peak fit with the estimate of *SF_H_*, the estimated number of hydrogen peak counts from the artillery shell is 31±5 counts per 10^8^ initial neutrons in the shell-point configuration.

## 4 Discussion

### 4.1 Signal optimization

The gamma flux incident on the detector of a PGNAA instrument is driven by the neutron flux at the unknown object and the geometry of the apparatus. In particular, care must be taken to minimize the distances between the neutron source, the unknown object and the detector, as both emissions processes, for neutrons and gamma rays, are isotropic (gammas) or nearly isotropic (neutrons).

Based on the studies described in Section 3.1, we conclude that a 50 mm thick disk of efficient neutron moderator material (polyethylene) is sufficient to provide a high thermal neutron flux to the unknown object while also maintaining a high fast neutron flux. Note that the features of the time distribution of thermal neutrons are sharper in the simulated data samples where the cavity is empty, such as those shown in Fig 4, than in those where it is filled with a dense unknown object such as the artillery shell. The thermalization that occurs within the unknown object’s volume softens the amplitude difference between the minimum and maximum of the time distribution.

Of the elements that are important to identify explosives and chemical weapons (H, N, As, F, P, S and Cl) [25], hydrogen is especially challenging. Its presence as a moderating agent is essential to generate a large flux of thermal neutrons. It is also a commonly occurring element, present in many of the materials used in the experimental apparatus. Care had to be taken in the design of the instrument to manage the background gamma-ray contribution from hydrogen-containing materials. Through iterative design, tested via simulation, we optimized choices of material and shielding to bring the hydrogen signal fraction *SF_H_* to a level that is compatible with a high precision analysis of an unknown object containing a high-explosive payload. Values of *SF_H_* at different stages of the optimization process are shown in Table 5. By doping the liquid shielding materials (water and oil) with boron to increase neutron absorption, and by substituting other materials for all non-essential polyethylene volumes, *SF_H_* increases from $2.8_{-0.4}^{+0.5}$%, a level at which long measurement times are necessary to ascertain the presence of signal from the object, to $19_{-4}^{+4}$%, for the shell-point design. This is already a significant improvement, however the value of *SF_H_* can be further increased to $53_{-7}^{+7}$% by adding lead shields around the detector to absorb gamma rays from sources other than the unknown-object. With *SF_H_* over or near 50% in this benchmark scenario of a 155 mm shell of TNT, identification of high explosives in most other unknown objects is expected to be possible. Further studies that include a variety of target objects are needed to precisely estimate the measurement times of both the benchmark case and other scenarios.

**Table 5:** Value of *SF_H_* in simulated event samples with the shell-point instrument design at various stages of the design optimization process.

Optimization stage	*SF_H_* [%]
Optimized neutron flux	$2.8_{-0.4}^{+0.5}$
Optimized materials	$19_{-4}^{+4}$
Optimized lead shielding	$53_{-7}^{+7}$
Optimized thermal-neutron time window	$74_{-10}^{+8}$

Fast neutrons dissipate from the unknown-object cavity within the first microsecond after neutron generation. As a result, the duration of the fast-dominant signal region depends entirely on the pulse length of the neutron generator. The pulse fall-off time of the generator design for this instrument is in the range 10–30 μs. Only a microsecond time gap is needed between the end of the pulse fall-off time and the start of data collection to ensure that the collected gamma-ray spectrum arises with high purity from thermal neutron interactions. The peak of the time distribution of thermal neutrons occurs approximately 100 μs after neutron creation, so a pulse duration between 100 and 300 μs should be used, in combination with the design operating frequency of a few kHz, to provide as many thermal neutrons as possible inside of the thermal-neutron time-window. Longer pulses would let the bulk of the thermal neutrons generated early in the pulse dissipate before signal begins to be recorded in the thermal-only window. However, the lifetime of thermal neutrons in the unknown-object cavity is of the order of milliseconds. As a result, a complementary time-window with a pure fast-neutron gamma-ray spectrum would require that the generator be operated at a frequency of not more than a few 100 Hz. To rapidly characterize an unknown object, a high neutron flux is needed, requiring a combination of high pulse frequency and intensity. A moderately high duty cycle would allow pauses of a few 100 μs, as required to maximize the signal from thermal neutron interactions. Pileup of thermal neutrons from earlier pulses is expected, but a fast-neutron dominant signal region can nonetheless be defined in the first few tens of μs at the beginning of each new pulse.

As is shown in Figs 11 and 14, after optimizing the instrument design, $SF_{H}=53_{-7}^{+7}$% (shell-point design) or $45.9_{-0.8}^{+0.8}$% (shell-side design), depending on the configuration used. The value of *SF_H_* increases to $74_{-10}^{+8}$% and $49_{-1}^{+1}$%, respectively, when limiting data acquisition to the time window within the thermal-only region (50 μs to next pulse) that maximizes *SF_H_* for detection of high explosives. Furthermore, limiting data acquisition to a thermal neutron-only window of interest reduces the number of counts at energies lower than 2.22 MeV in the gamma-ray energy spectrum by an order of magnitude (see Fig 13).

### 4.2 Measurement times

The hydrogen peak counts in the gamma-ray energy spectra from Section 3.5 can be used to estimate the measurement time requirements for hydrogen. For normal operation of the instrument, the anticipated neutron flux from the neutron generator is in the range of 1 − 5 × 10^9^ neutrons/s using a pulsing frequency of approximately 1 kHz, potentially reaching up to 1 × 10^11^ neutrons/s as the generator system improves [23]. With a generator flux of 1 × 10^9^ neutrons/s, we extrapolate from the fitted peak area of 64±11 counts per 10^8^ initial neutrons to estimate that 0.32 of data result in a 10% Poisson uncertainty contribution to the signal estimate. A 32 measurement would shrink this uncertainty to 1%. For a 30 minute measurement, it goes down to 0.13%. While there are small differences in the material budget and design of the shell-point instrument, for identical detectors, distance to the artillery shell is the main factor affecting the count rate. The centre of the detector volume is approximately 5 times further away from the centre of the neutron activation zone in the shell. The gamma-ray flux is reduced by a factor 25 based on distance, and the estimate of measurement time increases accordingly. The time needed to reach 1% Poisson uncertainty becomes approximately 15 minutes. The average detector count rates over the duration of the thermal-only window can also be estimated from the simulated hit energy spectra of Fig 15. It is (1.435±0.007)×10^5^ counts per second for the shell-side configuration and (2.6±0.1)×10^3^ counts per second in the shell-point configuration. The 10.8 nitrogen peak is small but it is in a low-background region of the spectrum, so once the number of signal events in the hydrogen peak has been determined, the H/N ratio for the shell content can be calculated from the spectrum using well-established PGNAA methods [26–28].

## 5 Conclusion

Monte Carlo simulations in GEANT4 were used to guide the design of a new active interrogation system for isotopic-content determination in artillery shells using a pulsed neutron generator. A 50 mm-thick neutron moderator disk is used in the instrument to maximize total neutron flux while generating a sufficient amount of thermal neutrons in the object interrogation cavity. Use of a pulsed neutron generator allows separation of the fast neutron-induced signal and the thermal neutron-induced signal, reducing background in both spectra. These two features allow for identification of high explosives using neutrons from the D-D reaction, which is not possible with currently available commercial instruments.

Two instrument designs have been optimized, one for the case where the gamma ray detector must be protected from the intense neutron flux from the generator, and one that can be used with detectors that are not affected by the presence of neutrons. The choice of material in many instrument parts was modified to minimize the presence of gamma-ray emissions from hydrogen in the gamma-ray spectrum measured by the detector. Furthermore, the design of the internal lead shielding was optimized. In the thermal-only time window, the hydrogen signal fraction reaches $45.9_{-0.8}^{+0.8}$% (Monte Carlo statistical uncertainty) in the “shell-side” configuration, which should be used whenever the detector system allows it, as the higher total gamma-ray flux enables more precise gamma-ray measurements for a given measurement time than in the “shell-point” configuration. In either configuration, 1% statistical precision in the determination of the hydrogen signal from a TNT-filled artillery shell can be achieved by a 30-minute measurement. A prototype instrument based on the shell-side design has been built and commissioning of the instrument has begun.

## Supporting information

S1 Text Readme. File containing format information and instructions to use the other files included as supporting information.
(TXT)

S1 File Archive file of data tables. All original data for the figures and results presented in this paper, tabulated. Each figure or subfigure corresponds to a file labeled with the figure’s number within the archive file.
(ZIP)
